# Annotations

**Published:** 1899-04-01

**Authors:** 


### ANNOTATIONS.

### The Microbe of Scarlet Fever.

In the last volume of the report of the medical officer to the Local Government Board there is a record of some observations made by Dr. Klein and Mr. Mervyn Gordon upon the microbes associated with scarlet fever which deserve more attention than they seem to have received. It is first pointed out that the streptococcus conglomeratus of Kurth is identical with the streptococcus scarletinæ which Dr. Klein some years ago isolated from the blood of acute cases of scarlet fever, and also from the ulcerations on the teats and udders of certain cows, the milk of which had been convicted of disseminating scarlet fever. It is stated that Kurth's contention that this streptococcus occurs constantly in the throat discharge of cases of acute scarlet fever has now, as the result of a considerable number of observations, been fully confirmed, and that its presence in the acutely inflamed throat of the human subject may be regarded as denoting scarlet fever just in the same way as, under corresponding throat conditions, the diphtheria bacillus is accepted as diagnostic of diphtheria. The fact that scarlet fever is thus authoritatively placed on the same footing as diphtheria so far as concerns bacteriological diagnosis is, of course, a matter of great interest; but perhaps even more so is the fact that, as is the case with diphtheria, the microbe can be discovered in the convalescent for a varying and, in some cases, for a very considerable period. It appears, according to Dr. Klein's investigations, that the streptococcus scarletinæ, which is always present in the throat secretion of persons acutely ill of scarlet fever, is even in the later stage of this malady, and after convalescence has been fully established, apt to be found in the throats of patients and sometimes in their nasal discharges, if the latter be present. Cases are mentioned in which it was found at intervals of six and nine weeks respectively from the date of the attack, and this even after the microbe had seemed to be absent from the secretions for a time Clearly this is a matter of considerable importance in relation to the question of when a patient is to be considered free from infection.

### An Acre of Land as an Old Age Pension.

Now that we hear so much about old age pensions it may not, perhaps, be uninteresting to draw attention to some remarks recently made on the subject by Dr. Vivian Poore, in the Milroy lectures. He said that agriculturalists were among the healthiest class in the community, and that even the farm labourer was 33 per cent. better than the average. He was often represented as half-starved, miserably housed, a martyr to rheumatism, and poisoned by filthy water. As a matter of fact, as regards sobriety and health, he might be taken as a model by the rest of the industrial classes. But there was another point of view from which agriculture was of great importance. We were beginning to find out that a factory hand who has to keep pace with steam machinery becomes "too old for his work" at a comparatively early age. Defects of eyesight or of hearing, or a lessening of acuteness or nimbleness, soon unfit a man for employments where dexterity is of more importance than experience. For such the land should be a great resource. Notwithstanding all that has of late been said about "old age pensions," it must be admitted, says Dr. Poore, that even if the financial difficulties of the question could be overcome, the prospect of being without employment and existing on a pittance without any true interest in life after the age of 55 years is not cheerful. "I believe," he continues, "that the practice of agriculture is the only remedy for this, and that the best old age pension will be found in the possession of an acre or so of land. Not only are the ordinary horticultural operations all possible for a man long after he is capable of attending to machinery, but such a possession would give him an interest in life." Whether the factory hand, or the worn out bootmaker or tailor, would care to go back to the land in his old age is another question. Probably he would not; but for all that we have a strong feeling of sympathy with what Dr. Poore says about the dull aimlessness of their latter days which is the lot of those who are driven from their work by the exigencies of trade rules, or perhaps of trades union rules, as soon as they become incapable of earning a young man's wage, and are thus condemned to throw away their experience and do "odd jobs" for the rest of their lives, or even, as some suggest, to live out a useless old age on a pension of five shillings a week. Wise is the man who takes to himself an occupation at which he can go on working so long as there is in him the capacity to work at all. According to Dr. Poore such an occupation is agriculture, with the possession of "an acre or so of land."

### Disinfection by Steam.

Some interesting experiments have recently been reported by Dr. Theodore Dunham to the Harvard Medical Society of New York in regard to the physics of sterilization by steam. The great point demonstrated was the obstructiveness of air to the rapid and complete diffusion of steam heat. It seems to have been too readily assumed that the pressure at which the steam gauge stands expresses the heat within the cylinder. As a fact, it does nothing of the sort. Unless the air is got rid of there is no assurance that all parts of the cylinder are raised to the required temperature. Steam is lighter than air, the air sinks to the bottom, and although the temperature in the upper part may be 122 deg. C. the lower part may never rise above 104 deg. C. Nor does it seem to be easy to overcome the difficulty by exhausting the air before the steam is admitted. The quickest way of getting a full diffusion of the heat is to let in the steam at the top and let the air go out at the bottom. Nevertheless, a preliminary exhaustion of the air is clearly a great advantage, especially in enabling the steam to obtain access to the inner recesses of bundles of material to be sterilised. This is not a matter of gravitation; the air is retained in the interstices of the materials by dint of what we may express as friction or adhesion, and unless the air is removed by exhaustion the steam cannot get in. The conclusion, then, is that for the general diffusion of the heat throughout the steriliser, the steam should come in at the top and the air should go out at the bottom; but that when it is required to make the steam penetrate masses of close material the air should be removed by exhaustion.

